# Rank correlation under categorical confounding

**DOI:** 10.1186/s40488-017-0076-1

**Published:** 2017-09-15

**Authors:** Jean-François Plante

**Affiliations:** Department of Decision Sciences, HEC Montréal, 3000 chemin de la Côte-Sainte-Catherine, Montréal, H3T 2A7 Canada

**Keywords:** Copulas, Rank statistics, Confounding, Weighted methods, MAMSE weights, 62H20, 62G05, 62G30, 62G10

## Abstract

Rank correlation is invariant to bijective marginal transformations, but it is not immune to confounding. Assuming a categorical confounding variable is observed, the author proposes weighted coefficients of correlation for continuous variables developed within a larger framework based on copulas. While the weighting is clear under the assumption that the dependence is the same within each group implied by the confounder, the author extends the Minimum Averaged Mean Squared Error (MAMSE) weights to borrow strength between groups when the dependence may vary across them. Asymptotic properties of the proposed coefficients are derived and simulations are used to assess their finite sample properties.

## Introduction

Correlation may be used to determine the strength of the link between two continuous variables. Rank correlation is often preferred as it makes no assumption on the marginal distributions of the variables and estimate their dependence structure directly. Those rank statistics are however not immune to the effect of confounding variables, and data with an underlying categorical variable may display a false correlation that is somewhat akin to an ecological fallacy when the marginal distributions differ between the groups implied by this confounder.

**Fig. 1:**
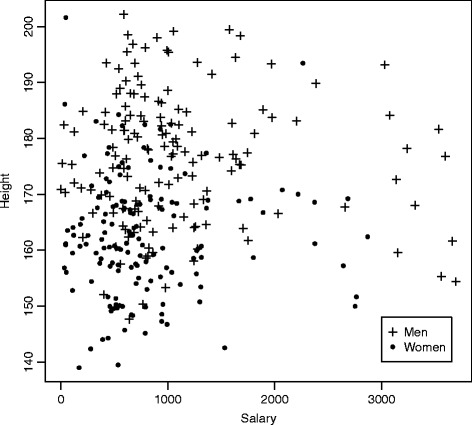
Scatter plot of height vs salary for 150 men and 150 women that are simulated as independent variables conditional on gender. A Spearman correlation of 0.137 (*p*-value=0.018) provides (false) evidence against independence

To illustrate, let us generate random data that show a spurious correlation between height and salary. Figure 1 displays a sample of 150 men and 150 women where the height and salary are generated independently, but their distributions depend on the gender. While the distribution of the height is based on the tables from Mc Dowell et al. (2008), the salary is generated to match statistics for weekly earnings from the Bureau of Labor Statistics. We make no attempt here at determining whether wages are equitable, we merely use factual distributions within a simplified simulation. Although Spearman correlations are -0.004 and 0.027 for the men and women respectively, the correlation calculated from the pooled samples amounts to 0.137 (*p*-value 0.018) due to the differences in the marginal distributions. Failing to take gender into consideration thus leads to wrongly concluding that salary and height are positively linked.

Differences in the marginal distributions across the groups defined by the confounding variable can be accounted for by calculating the ranks in these groups rather than globally. As a consequence, the sample is split in *m* smaller samples, and coefficients from each of these groups may be combined with appropriate weights. Under the assumption that the groups share the same dependence structure, any weighting will yield an unbiased estimate, but we also consider the case where the dependence in each group could differ. Whenever the dependence structure in the groups are likely to be similar, using data from all the groups wisely could provide a favorable tradeoff between bias and variance. Rather than keeping only the much smaller sample from each group of interest, we then suggest to use an extension of the Minimum Averaged Mean Squared Error (MAMSE) weights of Plante (2008, 2009a, b) to borrow strength adaptively from the other groups.

The dependence between two variables is best represented through the copula of their joint distribution. For continuous variables, the population value of rank correlation is a functional of that copula (see Genest and Nešlehová (2007) and Genest et al. (2014) for a descriptions of the challenges in the discrete case). While their original definitions are typically expressed as sums of ranks, coefficients of correlation based on ranks can also be rewritten as a functionnal of the empirical copula. We define MAMSE-weighted coefficients of correlation by replacing the empirical copulas in those alternative definitions with their MAMSE-weighted equivalent. In most cases, this is however equivalent to calcaulting a weighted sum of the coefficients of correlation.

Previous work on copula estimation in the presence of confounding variables includes Gijbels et al. (2011) and Veraverbeke et al. (2011) who use a form of kernel weighting based on a continuous confounder to estimate the marginal distributions as well as the copula underlying the data. Their approach is therefore based on similarities between the confounders, which is harder to define for a discrete variable. By comparison, the MAMSE weights are based on the similarities in the variables of interest between groups, which is possible because a discrete confounder provides a certain number of data for each level of the confounder. In this paper, we also have the notion of possibly homogeneous copulas with heterogeneous marginals which would not seem appropriate in the continuous setting treated by Gijbels et al. (2011) and Veraverbeke et al. (2011). The problem that we address requires a different approach than those proposed therein.

Background definitions and notation are provided in Section 2. Weights for empirical copulas are introduced in Section 3 as well as their theoretical properties. The same weights are used for coefficients of correlations based on ranks in Section 4 and convergence results are provided. Finally, Section 5 presents simulation results and a case study to illustrate the use of these weighted methods and explore their performance on finite samples. Technical proofs appear in the Appendix.

## Background and notation

We assume that a discrete finite confounding variable is observed along with *p*-dimensional continuous data of interest. For infinite variables, merging some values could offer a workaround, and if multiple discrete confounders are observed, they can be combined into one categorical variable through a cross-product. The complete sample is formed of independent variables, and is split in *m* different groups by the confounding variable. We use an index *k* to keep track of the simultaneously increasing sample sizes in the groups when studying asymptotic results. For any fixed *k*∈*IN*, we observe independent and identically distributed $\phantom {\dot {i}\!}\mathbf {X}_{i1},\ldots,\mathbf {X}_{in_{ik}} \sim F_{i}$ from Group *i*∈{1,…,*m*}, for a total of $\phantom {\dot {i}\!}N_{k}=\sum _{i=1}^{m} n_{ik}$ data. The observation $\phantom {\dot {i}\!}\mathbf {X}_{ij}=[X_{ij1},\ldots,X_{ijp}]^{\mathsf {T}}$ is a vector in *p* dimensions and *F*_*i*_ are continuous. By the theorem of Sklar (1959), there exists a unique copula *C*_*i*_ underlying the distribution *F*_*i*_ such that $\phantom {\dot {i}\!}F_{i}(\mathbf {x})=C_{i}\left \{G_{i1}(x_{1}),\ldots,G_{ip}(x_{p})\right \}$ where *G*_*i*1_,…,*G*_*ip*_ are the continuous marginal distributions of *F*_*i*_.

Let ${\mathbf {R}}^{k}_{ij}=\left [R^{k}_{ij1},\ldots,R^{k}_{ijp}\right ]^{{\mathsf {T}}}\phantom {\dot {i}\!}$ be the ranks associated with the vectors **X**_*ij*_, *j*=1,…,*n*_*ik*_. For fixed *i* and *ℓ*, the list of values $X_{i1\ell },\ldots,X_{in_{ik}\ell }\phantom {\dot {i}\!}$ is sorted and $R^{k}_{ij\ell }$ is the rank of *X*_*ijℓ*_ in that list. Since *F*_*i*_ are continuous, ties cannot occur with probability 1.

The empirical copula, ${\widehat{C}}_{ik}\left(\mathbf{u}\right)=\left(1/{n}_{ik}\right)\sum_{j=1}^{n_{ik}}\prod_{\ell =1}^p1\left({R}_{\mathrm{ij}\mathrm{\ell}}^k/{n}_{ik}\le {u}_{\ell}\right)$ with **u**=[*u*_1_,…,*u*_*p*_]^T^, uses ranks to estimate *C*_*i*_. The indicator variable *𝟙*(∙) is equal to one if its argument is true and equal to 0 otherwise. The empirical copula puts a weight of 1/*n*_*ik*_ on some points of an evenly spaced grid over [0,1]^*p*^ with exactly one such point in every (*p*−1)-dimensional slice of the grid (rows and columns in 2 dimensions).

For bivariate data, coefficients of correlation based on ranks measure concordance of the data. The population values of the well-known Spearman’s *ρ* and Kendall’s *τ* are $\rho =12\int uv \mathrm {d} C(u,v)-3$ and $\tau =4\int C(u,v) \mathrm {d} C(u,v)-1$ respectively, where *C* stands for the copula underlying the data. Substituting the empirical copula in these expressions leads to estimates that are asymptotically equivalent to the usual formulas (with *n* data having ranks (*R*_*i*_,*S*_*i*_) to adopt a simpler more common notation) $\hat {\rho }_{n}=-3(n+1)/(n-1)+12\{n(n+1)(n-1)\}^{-1}\sum _{j=1}^{n} R_{i}S_{i}\phantom {\dot {i}\!}$ and $\hat {\tau }_{n}=(2\{n(n-1)\}^{-1}\sum _{1\le i<j\le n}\text {sign}(R_{i}-R_{j})\,\text {sign}(S_{i}-S_{j})\phantom {\dot {i}\!}$. Both empirical coefficients are known to be asymptotically normal. Other measures of dependence such as Gini’s *γ* (Nelsen 1999) or Blest’s coefficients (Blest 2000; Genest and Plante 2003; Pinto da Costa and Soares 2005) are akin to Spearman’s *ρ* as they adopt the form of the expectation of a polynomial.

## Weights for mixtures of empirical copulas

Let ***λ***_*k*_=[*λ*_1*k*_,…,*λ*_*mk*_]^T^ be nonnegative weights such that $\sum _{i=1}^{m} \lambda _{ik}=1$ for all *k*∈*ℕ* and let

$$\hat{C}_{\boldsymbol{\lambda}_{k}}({\mathbf{u}})=\sum_{i=1}^{m} \lambda_{ik} \hat{C}_{ik}({\mathbf{u}}) $$

be a mixture of the empirical copulas based on the *m* available samples.

In this paper, inference must be made on the dependence between two or more variables (*C*_*i*_ or a functional thereof), conditional on the discrete confounding variable. The marginal distributions are therefore nuisance parameters. We look at two different situations where the dependence accross the groups is homogeneous or not. While it could be tempting to express equality of the dependence structure through coefficients of correlations, especially when it is the measure of interest, one has to remember that correlation does not fully determine dependence. Indeed, two sample can yield an equal correlation, say an equal Spearman’s *ρ*, but come from different copulas. Even with equal sample sizes, the variance of the estimates in these two samples will differ since their theoretical value depends on the true underlying copula (see e.g. Ruymgaart et al. 1972), not on the value of *ρ* alone. We consider the situation where all groups have a common dependence structure, and as such, it makes sense to assume equal copulas, i.e. *C*_1_=⋯=*C*_*m*_, rather than a weaker equality of the coefficients. The assumption of homogeneous dependence should be tested when required. We use a resampling procedure for that purpose in the case study. The second situation is when *C*_*i*_ differ between groups. Inference must then be made on each group individually since they do not have a common dependence structure. However, it is likely that although not equal, the dependence could be similar between many groups and we thus propose to use the MAMSE weights to borrow strength from other groups.

### 3.1 Homogeneous copulas: scalar weights

We first consider the paradigm where the *m* groups are assumed to share a common dependence structure, i.e. *C*_1_=⋯=*C*_*m*_=*C*. We allow for general scalar weights, but need Assumption 1 to ensure that each datum’s contribution tends to 0 as *k*→*∞*.

#### **Assumption 1**

We assume that $\lim \sup _{k} N_{k}/n_{ik}<\infty $ for *i*=1,…,*m* to ensure that all sample sizes increase at a similar rate. This also implies that $A_{k}=\sum _{i=1}^{m} \lambda _{ik}^{2} N_{k}/n_{ik}$ is finite for all *k*.

Deheuvels (1979) shows that $\sup _{{\mathbf {u}}\in [0,1]^{p}} |\hat {C}_{ik}({\mathbf {u}}) - C_{i}({\mathbf {u}})| \to 0$ almost surely as *k*→*∞* (since *n*_*ik*_→*∞* then). Similarly, the estimate $\hat {C}_{\boldsymbol {\lambda }_{k}}({\mathbf {u}})$ converges uniformly.

#### **Theorem 1**

$\sup _{{\mathbf {u}}\in [0,1]^{p}}|\hat {C}_{\boldsymbol {\lambda }_{k}}({\mathbf {u}})-C({\mathbf {u}})| \to 0$ almost surely as *k*→*∞*.

Let ${\mathcal {U}}_{i}({\mathbf {u}})$ be a *p*-dimensional centered Gaussian random field with covariance function *C*_*i*_(**u**∧**v**)−*C*_*i*_(**u**)*C*_*i*_(**v**), where ∧ is the component-wise minimum. Such a random field is called a *p*-dimensional pinned *C*_*i*_-Brownian sheet. Early results by Fermanian et al. (2004) and Tsukahara (2005) revisited by Segers (2012) (with weaker assumptions) show that $\sqrt {n_{ik}} \{\hat {C}_{ik}({\mathbf {u}}) - C_{i}({\mathbf {u}})\}$ converges weakly to such a Brownian sheet whose variance depends on *C*_*i*_ and its partial first-order derivatives. Since each of the $\hat {C}_{ik}({\mathbf {u}})$ are defined on independent samples and are asymptotically normal, the asymptotic distribution of $\hat {C}_{\boldsymbol {\lambda }_{k}}({\mathbf {u}})$ directly follows.

#### **Theorem 2**

The random variable $\sqrt {N_{k}/A_{k}} \{ \hat {C}_{\boldsymbol {\lambda }_{k}}({\mathbf {u}})-C({\mathbf {u}})\}$ converges weakly to the random field ${\mathcal {U}}({\mathbf {u}})-\sum _{\ell =1}^{p} \{(\partial /\partial u_{\ell }) C({\mathbf {u}})\} {\mathcal {U}}\left ([{\mathbf {1}},u_{\ell },{\mathbf {1}}]^{{\mathsf {T}}}\right)$ as *k*→*∞* where ${\mathcal {U}}({\mathbf {u}})$ is a random field with covariance structure *C*(**u**∧**v**)−*C*(**u**)*C*(**v**).

#### **Remark 1**

The choice of weights has an effect on the asymptotic distribution of the empirical copula. Simple calculus may be used to show that *λ*_*ik*_=*n*_*ik*_/*N*_*k*_ minimizes *A*_*k*_, hence yielding the least variable estimate $\hat {C}_{\boldsymbol {\lambda }_{k}}({\mathbf {u}})$. This choice corresponds to allocating an equal weight to each datum and yields *A*_*k*_=1. Since *λ*_*ik*_=*n*_*ik*_/*N*_*k*_ are optimal weights, all numerical examples involving scalar weights hereafter will be based on that choice of weights.

Note that our asymptotic paradigm involves a fixed number of groups whose sample sizes increase to infinity. The convergence would not hold for an infinite number of small groups. For instance, a mixture based on infinitely many samples of size 10 will still have $\hat {C}(1/20,1/20)=0$. Therefore, the convergence could fail if we were to increase the number of categories defined by confounding variables as the sample size increases.

Unless there are practical reasons to assume homogeneity of the copulas across groups, testing that assumption would seem advisable. Rémillard and Scaillet (2009) propose a test of equality between two copulas, but we need a test for a general number of groups *m*. Bouzebda et al. (2011) develop mathematical results for the *m*-sample empirical copula process, but while their results could lead to tests of equality for *m* samples, we could not locate a numerical implementation of such tests nor any results showing their finite sample properties. In the case study, we rather use resampling techniques to test the homogeneity of the copulas. Some basic properties of the proposed resampling algorithm are explored, but the future development of tests for the equality of the copulas in *m* samples will certainly provide better alternatives as they become available.

### 3.2 Heterogeneous copulas: adaptive weights

The assumption of identical dependence structures across groups may not always be appropriate. The problem to solve then becomes the inference of one or many of the *C*_*i*_. For simplicity, we will assume that only Group 1 is of interest, but the methodology developed could be applied sequentially to other groups of interest.

By identifying one group of interest, we adopt a paradigm similar to Wang and Zidek (2005) for the weighted likelihood. In this context, adaptive weights can trade potential bias for reduced variance. We therefore extend the MAMSE weights of Plante (2008, 2009a, b) by replacing the empirical distribution functions in their definition with empirical copulas.

Looking for a tradeoff between bias and variance means that the variance must play a role in the objective function that will determine the weights. Let us define

1 $$ P_{k}(\boldsymbol{\lambda})=\int_{[0,1]^{p}}\left[ \left|\hat{C}_{1k}({\mathbf{u}})-\hat{C}_{\boldsymbol{\lambda}_{k}}({\mathbf{u}})\right|^{2}+\sum_{i=1}^{m}\lambda_{i}^{2}\widehat{\text{var}} \left\{\hat{C}_{ik}({\mathbf{u}})\right\}\right]\mathrm{d} {{\mathbf{u}}}.  $$

While the first term in *P*_*k*_(***λ***) measures bias, the summation plays the role of a penalty for the variance that fosters using data from all the groups rather than limiting the inference to the group of interest. Since the asymptotic variance of the empirical copula depends on the true copula *C*_*i*_(**u**) and its derivatives, we consider a very rough estimate thereof given by

2 $$ \widehat{\text{var}}\{\hat{C}_{ik}({\mathbf{u}})\}\approx\widetilde{\text{var}}\{\hat{C}_{ik}({\mathbf{u}})\}= \frac{1}{n_{ik}} \hat{C}_{ik}({\mathbf{u}}) \{1-\hat{C}_{ik}({\mathbf{u}})\},  $$

which corresponds to the only term of the asymptotic variance of an empirical copula that does not involve a derivative of *C*_*i*_. The value of ***λ*** minimizing the objective function *P*_*k*_(***λ***) defined in (1) with the substitution (2) is called the MAMSE weights and is denoted ***μ***_*k*_. The algorithm for the MAMSE weights proposed by Plante (2008) implemented in the MAMSE R package can be used in the current context with copulas. Numerically, the integral is calculated on an evenly spaced grid with $n_{1k}^{p}$ points, or through Monte Carlo integration. Additional details specific to copulas may be found in Plante (2007).

The MAMSE weights have the property that $\int _{[0,1]^{p}} \left \{\hat {C}_{1k}({\mathbf {u}})-\hat {C}_{{\boldsymbol {\mu }}_{k}}({\mathbf {u}})\right \}^{2}\mathrm {d} {\mathbf {u}}\to 0$ almost surely as *k*→*∞*. Indeed, let ***λ***=[1,0,…,0]^T^ be a possibly suboptimal choice of weights for *P*_*k*_, and let ***μ***_*k*_ denote the MAMSE weights, then

3 $$ \int_{[0,1]^{p}} \left\{\hat{C}_{1k}({\mathbf{u}})-\hat{C}_{{\boldsymbol{\mu}}_{k}}({\mathbf{u}})\right\}^{2} \mathrm{d} {\mathbf{u}}\le P_{k}\{{\boldsymbol{\mu}}_{k}\}\le P_{k}(\boldsymbol{\lambda}) =\int_{[0,1]^{p}} \widetilde{\text{var}}\left\{\hat{C}_{1k}({\mathbf{u}})\right\}\mathrm{d} {\mathbf{u}}\le\frac{1}{4n_{1k}}.  $$

This property is key in proving Theorem 3, which would hold for other adaptive weights that respect the same condition.

#### **Theorem 3**

We have uniform convergence of the MAMSE-weighted empirical copula:

$$\sup_{{\mathbf{u}}\in[0,1]^{p}} \left|\hat{C}_{{\boldsymbol{\mu}}_{k}}({\mathbf{u}}) - C_{1}({\mathbf{u}})\right|\to 0 $$

almost surely as *k*→*∞*.

Note that the MAMSE weights display an irregular behaviour as *k*→*∞*. Although $\hat {C}_{{\boldsymbol {\mu }}_{k}}$ converges uniformly to the desired target, the rate of that convergence cannot be traced easily and the weights ***μ***_*k*_ may remain random for an arbitrarily large *k* if a mixture of the true *C*_2_,…,*C*_*m*_ is identical to *C*_1_. This behaviour is observed and discussed with other versions of the MAMSE weights in Plante (2008, 2009a). The study of the asymptotic distribution of $\sqrt {N_{k}}\left (\hat {C}_{{\boldsymbol {\mu }}_{k}}-C_{1}\right)$ would require a description of the similarities between the *C*_*i*_, an endeavour that will not be undertaken in this paper. Simulations and bootstrap can be used instead to determine the critical values for a test of hypothesis.

## Weighted coefficients of correlation

Many coefficients of correlation based on ranks including Spearman’s *ρ* (but not Kendall’s *τ*) take the form

4 $$ \hat{\kappa}_{ik}=a_{n_{k}}\int g({\mathbf{u}}) \mathrm{d} \hat{C}_{ik}({\mathbf{u}}) + b_{n_{k}}  $$

to estimate $\kappa _{i}=a\int g({\mathbf {u}}) \mathrm {d} C_{i}({\mathbf {u}}) + b$ where *g*(**u**) is a continuous bounded function on [0,1]^2^. The coefficients $a_{n_{k}}\to a$ and $b_{n_{k}}\to b$ as *n*_*k*_→*∞* are chosen to ensure that $\hat {\kappa }_{ik}\in [-1,1]$ for all sample sizes *n*_*ik*_ with the values ±1 occurring only for perfect concordance or discordance. Coefficients of the form $\hat {\kappa }_{ik}$ are asymptotically normal based on the results of Ruymgaart et al. (1972) and Ruymgaart (1974) (see also Genest and Plante (2003) for illustrations). Their variance can be derived from an expression that depends on the true copula underlying the data.

### 4.1 Homogeneous copulas

Assuming that *C*_*i*_=*C*, each $\hat {\kappa }_{ik}$ is normally distributed and it is clear that the random variable $\sqrt {N_{k}/A_{k}}\left (\hat {\kappa }_{\boldsymbol {\lambda }_{k}}-\kappa \right)$ converges weakly to a Normal variate with mean 0 and the same asymptotic variance as $\sqrt {n_{ik}}\left (\hat {\kappa }_{ik}-\kappa \right)$ when *k*→*∞*.

Coefficients of correlation are often used as a test of independence. Suppose that the alternative hypothesis is expressed through a parameter *θ* for which *θ*=0 yields independence. The theoretical value of *κ* is a function of *θ* and *κ*(0)=0. The asymptotic relative efficiency (*ARE*) of the two tests represent the ratio of the sample sizes needed by both tests to achieve the same power. To illustrate, suppose that we compare a test of independence based on $\hat {\kappa }$ with one based on Spearman’s $\hat {\rho }$. We find from Lehmann (1998), page 375, that $ARE\left (T_{\hat {\kappa }},T_{\hat {\rho }}\right)=\left (\sigma ^{2}_{\hat {\rho }}/\sigma ^{2}_{\hat {\kappa }}\right)\left (\kappa _{0}'/{\rho _{0}}'\right)^{2}$ where $T_{\hat {\kappa }}$ is the independence test based on $\hat {\kappa }$, *κ*0′=(*∂*/*∂* *θ*)*κ*(*θ*)|_*θ*=0_, $\sigma ^{2}_{\hat {\kappa }}$ is the asymptotic variance of $\hat {\kappa }$, and similarly for $\hat {\rho }$.

#### **Remark 2**

If the marginal distributions were not affected by the confounder, we could pool the *N*_*k*_ data together to yield a test based on the usual estimate $\hat {\kappa }$ calculated on the whole dataset. In that case, $ARE\left (T_{\hat {\kappa }},T_{\hat {\kappa }_{\boldsymbol {\lambda }_{k}}}\right)={\lim }_{k\to \infty }A_{k}$. Recall that *A*_*k*_=1 when *λ*_*i*_=*n*_*ik*_/*N*_*k*_, which means that there is no loss of power asymptotically for using a weighted coefficient.

Let us also consider the estimate $\hat {\tau }_{\boldsymbol {\lambda }_{k}}\,=\,\sum _{i=1}^{m} \lambda _{i} \hat {\tau }_{ik}$. Since $\hat {\tau }_{ik}$ is a *U*-statistics, $\sqrt {n_{ik}}(\hat {\tau }_{ik}\!-\tau)$ is asymptotically distributed as a centered Normal variable, hence $\sqrt {N_{k}/A_{k}}(\hat {\tau }_{\boldsymbol {\lambda }_{k}}-\tau)$ converges weakly to a Normal distribution under the assumption that the copulas of the *m* groups are equal.

### 4.2 Heterogeneous copulas

When copulas are not assumed equal across groups, the MAMSE weights may be used to define consistent coefficients of correlation. Recall that within this paradigm, the dependence of each group is assessed individually while borrowing strength from the other groups. To simplify presentation, only Group 1 is deemed of interest, but the same methodology could be applied sequentially to every group if needed.

#### **Theorem 4**

Coefficients $\hat {\kappa }_{{\boldsymbol {\mu }}_{k}}$ defined with a function *g*(**u**) bounded on [0,1]^*p*^ are strongly consistent, i.e. $ \hat {\kappa }_{{\boldsymbol {\mu }}_{k}}\to \kappa _{1}$ almost surely as *k*→*∞*.

One strength of the MAMSE weights is that almost no assumptions are made about the underlying distributions in the *m* groups, yet consistency is secured. Determining rates of convergence and the asymptotic distribution of MAMSE based statistics would however require much stronger assumptions about the relative shape of the distributions in the *m* groups. For testing and inference, we prefer to rely on resampling methods.

Let us now consider $\hat {\tau }_{{\boldsymbol {\mu }}_{k}}=\sum _{i=1}^{m} \mu _{i} \hat {\tau }_{ik}$. With heterogeneous copulas, the lack of linearity of *τ* may cause $\hat {\tau }_{{\boldsymbol {\mu }}_{k}}$ to be inconsistent.

#### **Remark 3**

Consider the Fréchet family of copula from Example *5.3* in Nelsen *(* 1999 *)*, page *129*. *C*_1_=*C*_*α*,*β*_=*α* *M*+(1−*α*−*β*)*Π*+*β* *W* where *M*=*C*_2_, *Π*=*C*_3_ and *W*=*C*_4_ represent respectively the Fréchet bounds of perfect concordance, independence and perfect discordance. In this situation, the adaptive weights will find ***μ***_*k*_ such that $C_{{\boldsymbol {\mu }}_{k}}\to C_{1}\phantom {\dot {i}\!}$, but the share of *C*_1_ compared to *C*_2_, *C*_3_ and *C*_4_ may remain random even for large *k*. Unfortunately, *τ*_1_=(*α*−*β*)(*α*+*β*+2)/3 is not equal to *α* *τ*_*M*_+(1−*α*−*β*)*τ*_*Π*_+*β* *τ*_*W*_=*α*−*β*, meaning that $\hat {\tau }_{{\boldsymbol {\mu }}_{k}}$ will not be consistent, and may in fact not even converge to a single value.

The empirical version of Kendall’s *τ* can be written as $\hat {\tau }_{ik}=4n(n-1)^{-1} \int \hat {C}_{ik}({\mathbf {u}}) \mathrm {d}\hat {C}_{ik}({\mathbf {u}})-\left \{1+4(n-1)^{-1}\right \}$, which shows that Kendall’s *τ* is asymptotically equivalent to replacing the copula by its empirical counterpart in the population value of *τ*. We thus define a new statistic based on $4\int \hat {C}_{{\boldsymbol {\mu }}_{k}}({\mathbf {u}}) \mathrm {d} \hat {C}_{{\boldsymbol {\mu }}_{k}}({\mathbf {u}})-1={\boldsymbol {\mu }}_{k}^{{\mathsf {T}}} \hat {\mathbf {\mathcal {T}}}_{k}{\boldsymbol {\mu }}_{k}$ where $\hat {\mathbf {\mathcal {T}}}_{k}$ is a *m*×*m* matrix with $[\hat {\mathbf {\mathcal {T}}}_{k}]_{ij}=4\int \hat {C}_{ik}({\mathbf {u}}) \mathrm {d} \hat {C}_{jk}({\mathbf {u}})-1$. To facilitate their interpretation, coefficients of correlation are usually built to have a null expectation under independence. In addition, under perfect negative or positive dependence, the coefficients take values -1 and 1 respectively. We define the asymptotically equivalent expression

$${\begin{aligned} \widetilde{\tau}_{{\boldsymbol{\mu}}_{k}}={\boldsymbol{\mu}}_{k}^{{\mathsf{T}}} \widetilde{{\mathbf{\mathcal{T}}}}_{k}{\boldsymbol{\mu}}_{k}\qquad\text{where}\qquad\left[\widetilde{\mathbf{\mathcal{T}}}_{k}\right]_{ij}=\frac{1}{N_{ijk}}\sum_{s=1}^{n_{ik}} \sum_{t=1}^{n_{jk}} \text{sign}\left(\frac{R^{k}_{is}}{n_{ik}}-\frac{R^{k}_{jt}}{n_{jk}}\right) \,\text{sign}\left(\frac{S^{k}_{is}}{n_{ik}}-\frac{S^{k}_{jt}}{n_{jk}}\right) \end{aligned}} $$

and $$ {N}_{ijk}=\sum_{s=1}^{n_{ik}}\sum_{t=1}^{n_{jk}}1\left({R}_{is}^k/{n}_{ik}\ne {R}_{jt}^k/{n}_{jk}\right)1\left({S}_{is}^k/{n}_{ik}\ne {S}_{jt}^k/{n}_{jk}\right) $$ is such that $\left [\widetilde {{\mathbf {\mathcal {T}}}}_{k}\right ]_{ii}=\hat {\tau }_{ik}$. Although $\left [\widetilde {{\mathbf {\mathcal {T}}}}_{k}\right ]_{ij}=1$ under perfect positive dependence, we rather get $\mathrm {E}_{\Pi }\left (\left [\widetilde {{\mathbf {\mathcal {T}}}}_{k}\right ]_{ij}\right)= \left (n_{ik}n_{jk}/N_{ijk}\right)\left (1/n_{jk}-1/n_{ik}\right)^{2}$ under the assumption of independence and an unwieldy expression for negative dependence. In general, $\mathrm {E}_{\Pi }\left (\left [\widetilde {{\mathbf {\mathcal {T}}}}_{k}\right ]_{ij}\right)$ is not the mid-point of the values of $\widetilde {{\mathbf {\mathcal {T}}}}_{k}$ under perfect positive and negative dependence, except if *n*_*ik*_=*n*_*jk*_. As a consequence, even a linear transformation cannot make $\widetilde {\tau }_{{\boldsymbol {\mu }}_{k}}={\boldsymbol {\mu }}_{k}^{{\mathsf {T}}} \widetilde {{\mathbf {\mathcal {T}}}}_{k}{\boldsymbol {\mu }}_{k}$ fit the magical values of -1, 0 and 1 appropriately for finite samples, contrarily to the inconsistent $\hat {\tau }_{{\boldsymbol {\mu }}_{k}}$ who preserves this property.

#### **Theorem 5**

$\widetilde {\tau }_{{\boldsymbol {\mu }}_{k}}\to \tau _{1}$ almost surely as *k*→*∞*.

The asymptotic normality of $\hat {\tau }$ can be derived from the theory on *U*-statistics, but $\widetilde {\tau }_{{\boldsymbol {\mu }}_{k}}$ does not fall within this paradigm. Resampling methods may be used for testing or establishing confidence intervals.

## Simulations and case study

Simulations are used to explore the finite-sample performances of the proposed weighted methods under different scenarios. Scalar weights that are proportional to the sample sizes (***λ***) are considered as well as the adaptive MAMSE weights (***μ***). Note that the index *k* is dropped in this section where sample sizes are fixed. The R package *MAMSE* from the Comprehensive R Archive Network offers functions to compute the MAMSE weights as well as weighted coefficients of correlation.

### 5.1 Salary vs height

We first revisit the example presented in the introduction where salary and height are simulated as independent variables, but with different marginal parameters for men and women. Height is simulated as a Normal variable with mean 176.3 cm and standard deviation 11.38 cm for men, but mean 162.2 cm and standard deviation 11.15 for women. Those parameters are based on the tables from Mc Dowell et al. (2008). Salary is simulated based on quantiles or order 10, 25, 50, 75 and 90% (average value over quarters of 2009 for each quantile) for the usual weekly earnings of men and women as calculated by the Bureau of Labor Statistics (accessed online at http://www.bls.gov/webapps/legacy/cpswktab5.htm). The salary are assumed to be uniformly distributed between the given quantiles. For the purpose of the simulation, it is assumed that the minimum salary is 0 and that the maximum salary equals twice the 90th quantile. No attempt is made here to study the possibility of wage inequity: we barely use the distributions of height and salary to illustrate the potential effect of a discrete confounder when marginal distributions are nuisance parameters. The simulation described is repeated 10,000 times.

**Fig. 2:**
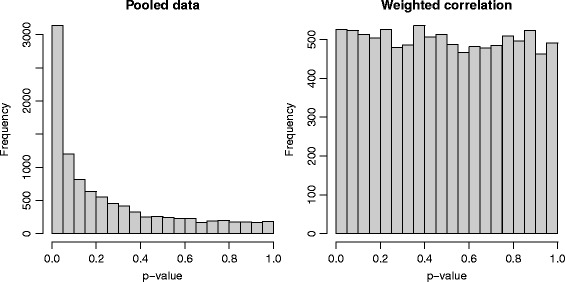
Histograms of 10,000 *p*-values of a test of independence. Salary and Height are simulated as independent random variables with gender-specific marginal distributions. On the left panel, the tests of independence are based on Spearman’s rho calculated on the pooled data. On the right panel, a weighted version of the coefficient of correlation leads to an apparently unbiased test

Figure 2 shows the p-values of tests of independence based on Spearman’s rho. If we consider a 5% level for a test, ignoring potential differences between men and women leads to a 31.4% rejection rate. The weighted coefficient $\hat {\rho }_{\boldsymbol {\lambda }}$, however, provides and unbiased test with an observed 5.3% rejection rate and an histogram that approaches a uniform distribution as expected.

### 5.2 Case study: the Iris dataset

Consider now the famous Iris dataset from Fisher (1936). The variables are respectively sepal length, sepal width, petal length and petal width, all measured in centimeters, for 50 Iris Setosa, 50 Iris Versicolor and 50 Iris Virginica. Although a trained eye would not mistake them for one another, these three species of flowers are relatively similar in color and shape. Looking at the correlation between the measurements may give an idea of the geometry of the flowers. For instance, do the petals of larger specimen keep the same shape, which would translate in a highly positive correlation between their length and width.

**Fig. 3:**
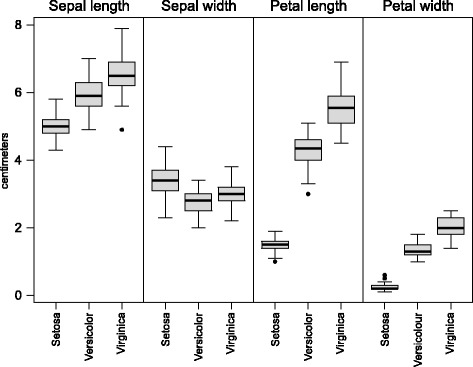
Side-by-side boxplots for the marginal data of the Iris data set by species

The descriptive analysis of the marginal distributions found in Fig. 3 shows that the species have different marginal characteristics. A correlation that does not take into consideration this confounding variable therefore presents a biased picture of reality. To fix ideas, Table 1 displays estimates of the Spearman correlation matrix for the whole data set ($\hat {\boldsymbol {\rho }}$) on the left, then the same matrix based only on the data of each group ($\hat {\boldsymbol {\rho }}_{i}$). While the three groups have generally similar types of correlations structures, $\hat {\boldsymbol {\rho }}$ offers a spurious picture that includes negative correlations. Beyond the general picture, the correlations matrices of the three groups display clear discrepancies: it is unlikely that the assumption of homogeneous copulas could hold and support a preference for $\hat {\rho }_{\boldsymbol {\lambda }}$. As a matter of fact, a resampling test based on 10,000 bootstrap samples gave a p-value of 0. Details of the resamlping methods can be found in the next subsection. Since $\hat {\rho }_{\boldsymbol {\lambda }}$ would offer a biased view, we are left with the MAMSE-weighted coefficients of correlation to estimate each ***ρ***_*i*_ separately, or to rely only on data from Group *i* to estimate ***ρ***_*i*_.

**Table 1:** Spearman’s correlation matrices for sepal length, sepal width, petal length and petal width

$ \hat {\boldsymbol {\rho }}=\left [\begin {array}{rrrr} 1&&&\\ -0.16&1&&\\ 0.88&-0.30&1&\\ 0.83&-0.28&0.94&1\\ \end {array}\right ]$	$\hat {\boldsymbol {\rho }}_{1}=\left [\begin {array}{rrrr} 1&&&\\ 0.77&1&&\\ 0.27&0.18&1&\\ 0.30&0.37&0.23&1\\ \end {array}\right ]$	$\hat {\boldsymbol {\rho }}_{2}=\left [\begin {array}{rrrr} 1&&&\\ 0.52&1&&\\ 0.74&0.57&1&\\ 0.55&0.66&0.79&1\\ \end {array}\right ]$	$\hat {\boldsymbol {\rho }}_{3}=\left [\begin {array}{rrrr} 1&&&\\ 0.43&1&&\\ 0.82&0.39&1&\\ 0.32&0.54&0.36&1\\ \end {array}\right ]$

While $\hat {\boldsymbol {\rho }}_{i}$ contain Spearman’s coefficients for each each of the three species of iris, namely Setosa ($\hat {\boldsymbol {\rho }}_{1}$), Versicolor ($\hat {\boldsymbol {\rho }}_{2}$) and Virginica ($\hat {\boldsymbol {\rho }}_{3}$), the matrix $\hat {\boldsymbol {\rho }}$ contains Spearman’s correlation for the 150 iris taken as a single dataset, hence ignoring marginal discrepancies

To estimate the correlations for, say Iris Versicolor, one could calculate the MAMSE weights on the four dimensional data and combine the $\hat {\boldsymbol {\rho }}_{i}$ matrices accordingly. These weights are determined based on the similarities of four-dimensional empirical copulas across the three groups and must therefore strike a global compromise. If for instance the dependence of petal width and length is very similar across groups, but correlations involving the sepals are much less akin, they will still all be combined with the same weights. An alternative approach is to consider every pair of variables, and to compute bivariate MAMSE weights for them. The adaptation to similarities is improved, but there is no guarantee that the resulting matrix is positive definite. If interest lays in the correlations rather than the correlation matrix, this may be a better option.

In order to evaluate the performance of the different methods, we now run a simulation where parameters obtained from the Iris dataset are assumed to be the “true model”. Using the R package *MVN*, we find that Mardia’s test does not reject multivariate normality of the three datasets from each species of iris. We will therefore draw simulated iris from a multivariate normal distribution with parameters equal to the estimated mean and covariance obtained from the original Iris dataset. Knowing the “real” distribution of the data will allow to evaluate the Mean Squared Error (MSE) of each estimate.

We generate 10,000 samples of 150 iris, 50 of each species, and we compute Spearman’s correlation based on the two types of MAMSE weights. While the “global” weights are based on the four-dimensional data, the “pairwise” weights are determined separately for each group of two variables, allowing for increased flexibility. Each of the three species of iris are considered, in turn, as the target distribution. Table 2 shows $100\,MSE(\hat {\rho }_{i})/MSE(\hat {\rho }_{{\boldsymbol {\mu }}})$, the relative MSE comparing a version of MAMSE to its competitor based solely on the group of interest. The relative MSE is reported for each pairwise correlation for both global and pairwise weights. For the global weights, the MSE of the correlation matrices corresponds to the average MSE for each coefficient of that matrix and is also reported as a relative measure.

**Table 2:** Relative MSE of Spearman’s correlation matrices for sepal length (SL), sepal width (SW), petal length (PL) and petal width (PW)

			SL			SW		PL
Species of interest	MAMSE	Matrix	SW	PL	PW	PL	PW	PW
Setosa	Global	76	34	38	170	103	92	132
	Pairwise		25	38	174	77	68	104
Versicolor	Global	170	139	120	250	175	169	272
	Pairwise		154	139	350	225	217	284
Virginica	Global	181	208	207	159	140	149	79
	Pairwise		198	169	169	136	141	95

The values listed are $100\,MSE(\hat {\rho }_{i})/MSE(\hat {\rho }_{{\boldsymbol {\mu }}})$ and are based on 10,000 repetitions. Each species of iris is in turn the target group. The MAMSE weights are calculated based on a global or pairwise strategy. Relative MSE are reported for each pairwise correlation, as well as for the correlation matrix in the case of global weights

We first note that the MAMSE weights provide improved performance in most cases. The estimation of the correlation for Iris Versicolor, for instance, is always better with a MAMSE-weighted correlation, and the pairwise approach is systematically best. For Iris Virginica, both MAMSE approaches seem acceptable since they provide improved performance everywhere, except for the correlation between petal length and petal width. At the other end of the spectrum, the MAMSE-weighted correlations sometimes show weaker performances as it is the case for Iris Setosa. Looking at Table 1 we may notice that $\hat {\boldsymbol {\rho }}_{1}$ is the correlation matrix that seems the most dissimilar to the other ones. While an infinite sample size would still guarantee an efficient estimate, there are cases where a loss of efficiency is observed for finite samples. Such observations are also made by Plante (2008) who describes in the univariate case how the MAMSE weights initially boost the performance for small samples, and provides equivalent performance for very large samples. In between, there is often a certain range for which the MAMSE approach does not offer an improved performance. Note also that although estimation for Iris Setosa was not improved by the contribution of the other two kinds of iris, using Iris Setosa to estimate the parameters of the other two types of iris did yield better performances. In the MAMSE objective function, any bias must be compensated by an equally reduced variance, but transformation of the copulas into other statistics may change the geometry of bias and variance. The MAMSE weights do not provide a uniformly more efficient approach, but overall, it seems to offer an appreciable gain.

In this example, we ran a simulation inspired from a real dataset. That approach could be considered by somebody who wonders how much improvement they could expect from the MAMSE approach: They could run a simulation on a model that mimics their own data.

### 5.3 Resampling technique for testing the homogeneity of copulas

To test homogeneity of the copulas, we need a nonparametric test for the equality of copulas in *m* groups. As mentioned in Section 3.1, Rémillard and Scaillet (2009) have developped a solution for *m*=2 and the results from Bouzebda et al. (2011) for more groups have not been implemented numerically nor tested on finite samples. While further developments of such tests will certainly offer better options in the near future, we choose here to use a resampling method that we present next.

The test is based on a Cramér-Von-Misses type statistic, namely

$$T=(1/m)\sum_{i=1}^{m} \int_{[0,1]^{p}}\left\{\hat{C}_{ik}({\mathbf{u}})-\hat{C}_{-ik}({\mathbf{u}})\right\}^{2}\,\mathrm{d} {\mathbf{u}} $$

where $\hat {C}_{-ik}({\mathbf {u}})=\sum _{j\neq i} \{n_{j}/(N-n_{i})\}\hat {C}_{jk}({\mathbf {u}})$ is a mixture based on all groups except for *i*. In our implementation for this paper, Monte Carlo integration (with 2000 random points) is used to evaluate the integrals in the Cramér-Von-Misses statistic. The resampling test follows these steps:

1. Calculate the ranks ${\mathbf {R}}^{k}_{ij}$ on the raw data (the ranks are taken within each groups) and rescale them by dividing by *n*_*i*_. This step gets rid of the (nuisance) marginal distributions.
2. Pool the rescaled ranks $\left ({\mathbf {R}}^{k}_{ij}/n_{i}\right)$ into a single set. Under the null hypothesis of homogeneity of dependence, those groups of rescaled ranks all follow (approximately) the same copula common to the groups.
3. Generate bootstrap samples of size *n*_1*k*_,…,*n*_*mk*_ by drawing witout replacement from the pooled list of ranks.
4. Calculate the ranks in each bootstrap sample, then compute the Cramér-Von-Misses type statistic presented above.
5. Calculate the same Cramér-Von-Misses type statistic on the original data. If it is bigger than the 95% bootstrap quantile, then homogeneity is rejected. Alternatively, a p-value is obtained by taking the proportion of bootstrap samples yielding a statistic greater than or equal to the statistic computed on the original data.

**Fig. 4:**
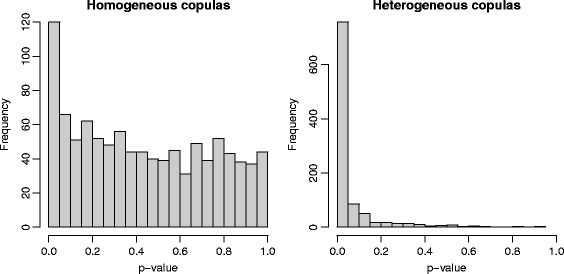
Histograms of 1000 *p*-values of a resampling test for the homogeneity of the copulas. Simulated iris datasets are generated from two scenarios. While on the left panel, the three species of iris share a same copula, on the right panel, the three species are generated as a multivariate normal with parameters estimated from the three species of iris in the original dataset

To confirm that this test has reasonnable finite sample properties, we ran a small simulation for scenarios inspired from the Iris dataset. We simulated 3 groups of 50 data from the normal distributions described in the previous section. To represent homogeneity of the copulas, the parameters of Iris Setosa were used for the three groups in a first scenario. A second scenario used the parameters from the original dataset, thus different covariances in each group. In both cases, 1000 samples (of three times 50 iris) were generated and for each, 1000 bootstrap samples were used in the resampling method to determine a *p*-value for the test of homogeneity. Figure 4 shows the histograms of those *p*-values.

Under the null hypothesis, the test appears to be conservative with a histogram that displays too many small values. As a matter of fact, a 5% level test would have rejected the null with probability 0.120. Resampling from ranks creates ties which could explain that bias. The right panel of Fig. 4 shows that the test has reasonnable power in the context of the Iris dataset. The same 5% level test has a power of 0.757 under that heterogeneous scenario.

### 5.4 Homogeneous copula

This simulation is designed to measure the loss of efficiency that is suffered when using the proposed weighted methods. Throughout this section, the benchmark method is to pool all the data in a single set, an impossible endeavour with real data because of the confounding.

Theoretical results showed that the scalar-weighted rank statistics considered are unbiased and that their asymptotic variance is not affected by the splitting of the sample in *m* groups when optimal scalar weights are used, but is there a measurable loss on finite samples? The MAMSE-weighted statistics are consistent, but they could be biased on finite samples. How much do we lose for not assuming homogeneity of the dependence when that assumption is in fact true?

We generate data with homogeneous copulas from a Clayton distribution (Clayton^1978^ *; Nelsen* 1999) whose parameter is set to yield a Spearman’s correlation of *ρ*∈{0.1,0.5,0.9}. A total of 5*n* data points are available as samples of equal sizes from 5 groups. For each value of *ρ* and *n*∈{10,20,50}, 10,000 sets of samples are simulated and homogeneity of the copulas is assumed without being tested. Although it would not be possible to pool the data into a single set in a real case because of the nuisance marginal distributions, we use that situation as an unreachable benchmark. The estimates based on pooled data can be recognized by their lack of index (they are noted $\hat {C}$, $\hat {\rho }$ and $\hat {\tau }$).

To evaluate the precision of the weighted empirical copula $\hat {C}_{\boldsymbol {\lambda }}$, the upper section of Table 3 shows the ratio $100\,\int |\hat {C}({\mathbf {u}})-C({\mathbf {u}})|\mathrm {d}{\mathbf {u}} / \int |\hat {C}_{\boldsymbol {\lambda }}({\mathbf {u}})-C({\mathbf {u}})|\mathrm {d}{\mathbf {u}}$, and similarly for $\hat {C}_{{\boldsymbol {\mu }}}$. The theoretical results about $\hat {C}_{\boldsymbol {\lambda }}$ mention that it is unbiased and has the same asymptotic variance as $\hat {C}$. Surprisingly, this conservation of the efficiency is visible even for a samples as small as *n*=10, and for all strengths of correlation. Estimating the MAMSE weights has a cost, so a smaller efficiency is expected for $\hat {C}_{{\boldsymbol {\mu }}}$, but the loss is fairly small.

**Table 3:** Performance of different weighted measures of dependence reported as $100 \int |\hat {C}({\mathbf {u}})- C({\mathbf {u}})|\mathrm {d}{\mathbf {u}} / \int |\hat {C}_{\boldsymbol {\lambda }}({\mathbf {u}})-C({\mathbf {u}})|\mathrm {d}{\mathbf {u}}$ or by a ratio of the kind $100\,\text {MSE}(\hat {\rho })/\text {MSE}(\hat {\rho }_{\boldsymbol {\lambda }})$

	*ρ*=0.1			*ρ*=0.5			*ρ*=0.9		
	*n*=10	20	50	*n*=10	20	50	*n*=10	20	50
$\hat {C}_{\boldsymbol {\lambda }}$	100	100	100	100	100	100	100	100	100
$\hat {C}_{{\boldsymbol {\mu }}}$	93	93	93	94	95	95	99	99	99
$\hat {\rho }_{\boldsymbol {\lambda }}$	93	94	98	78	87	95	35	48	69
$\hat {\rho }_{{\boldsymbol {\mu }}}$	60	64	67	53	63	72	33	46	68
$\hat {\tau }_{\boldsymbol {\lambda }}$	79	86	95	76	86	95	66	77	91
$\hat {\tau }_{{\boldsymbol {\mu }}}$	52	59	65	54	64	72	61	73	88
$\widetilde {\tau }_{{\boldsymbol {\mu }}}$	45	56	63	46	59	70	39	50	72

In a practical situation, the confounding would make it impossible to calculate $\hat {C}$, $\hat {\rho }$ and $\hat {\tau }$ on the whole dataset, but they are used here as unattainable ideal benchmarks. Five samples of size *n* are simulated from a Clayton distribution with Spearman’s correlation *ρ*. Each scenario is repeated 10,000 times

Weighted coefficients of correlation are also calculated on the samples described above. Their performance measured by ratios such as $100\,\text {MSE}(\hat {\rho })/\text {MSE}(\hat {\rho }_{\boldsymbol {\lambda }})$ appear in the lower part of Table 3. Note that without the proposed methodology, the alternative would be to use only Group 1 for inference, which would yield a ratio of 20. Compared to that achievable benchmark, the weighted methods always provide an improvement. While $\hat {\rho }_{\boldsymbol {\lambda }}$ is asymptotically efficient, its efficiency is not attained on small samples, but clearly increases as *n* increases. Remember that in a real life setting, the confounding makes it impossible to compute $\hat {\rho }$ directly, so the loss of efficiency observed may be unavoidable. Although we did not compute its ARE explicitly, $\hat {\tau }_{\boldsymbol {\lambda }}$ shows a behaviour similar to $\hat {\rho }_{\boldsymbol {\lambda }}$. This good behaviour is not surprising under homogeneous copulas since $\hat {\tau }_{\boldsymbol {\lambda }}$ is then unbiased with a variance equal to that of $\hat {\tau }$. The performance of the weighted coefficients of correlation seem to decrease as the correlation gains in strength. Splitting the dataset in multiple smaller samples reduced the variety of values that an empirical coefficient may achieve and this becomes more acute with larger correlations as most combinations of ranks become improbable. In general, using the MAMSE weights when the copulas are homogeneous decreases the performance, but we can observe that the loss is reasonable. The MAMSE weights are clearly offering a better performance while protecting against heterogeneity.

When considering Kendall’s *τ*, the weighted avaverage $\hat {\tau }_{\boldsymbol {\lambda }}$ performs best. In the homogeneous case, this coefficient could be considered. Even if $\hat {\tau }_{{\boldsymbol {\mu }}}$ shows better performances than $\widetilde {\tau }_{{\boldsymbol {\mu }}}$, we would still recommend the latter in the heterogeneous case given that $\hat {\tau }_{{\boldsymbol {\mu }}}$ may not be consistent.

### 5.5 Tests of independence

Remark 2 mentions that there is no asymptotic loss of power in testing independence using a scalar-weighted version of Spearman’s *ρ*. To obtain a more complete picture, we study the power of tests of independence based on different coefficients of correlation, including weighted coefficients with scalar and MAMSE weights. Five groups of equal size *n*=20 are simulated from a Clayton copula under three scenarios where the parameter of the Clayton is matched to Spearman’s *ρ* and expressed as such (even for simulations about *τ*). The true correlation in Group *i* is therefore noted *ρ*_*i*_. Power graphs are plotted as a smoothed line based on 51 different values of *ρ*_1_, and for each of these values of the graph, 1000 repetitions of a test of independence are generated. The first scenario has homogeneous copulas (*ρ*_1_=*ρ*_2_=*ρ*_3_=*ρ*_4_=*ρ*_5_) hence the scalar weights should be performing optimally. In the other scenarios, groups 2 to 5 have a fixed correlation while only *ρ*_1_ varies according to the x-axis. In Scenario 2 *ρ*_2_=*ρ*_3_=*ρ*_4_=*ρ*_5_=0.1, but in Scenario 3, *ρ*_2_=−0.4, *ρ*_3_=−0.2, *ρ*_4_=0.2, *ρ*_5_=0.4.

To test independence with a sample of size *n*, Spearman’s *ρ* is compared to a centered Normal with variance 1/(*n*−1) and Kendall’s *τ* to a centered Normal with variance (4*n*+10)/{9*n*(*n*−1)} (see e.g. Capéraà and Van Cutsem^1988^). For weighted coefficients based on five groups with equal scalar weights, the same formulas are used with the total sample size. Tests based on the MAMSE-weighted coefficients are trickier as the weights depend not only on Group 1, but also on data from the other groups. In particular, it depends on data that are not covered by the tested hypothesis *H*_0_:*ρ*_1_=0. To determine if one should reject the null or not, we therefore proceed with resampling techniques where a new sample is generated for Group 1 from the independence copula while sampling with replacement is applied to each of the other groups. To keep the computations manageable, each test is based on 400 bootstrap samples that are use to determine the standard error of the MAMSE-weighted coefficients of correlation. A Wald-type statistic is then used to test independence.

**Fig. 5:**
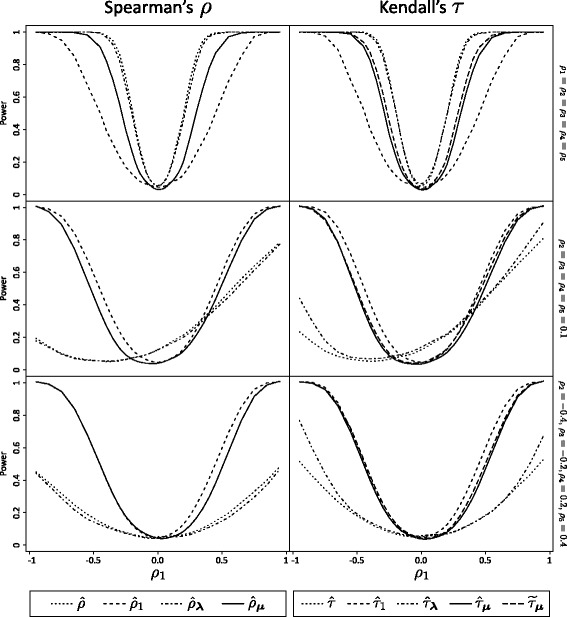
Power of a test of independence based on different coefficient of correlations. The two columns of plots are respectively for estimates of Spearman’s *ρ* and Kendall’s *τ*, the rows correspond to different scenarios described with equations on the right. Equal samples of size *n*=20 are drawn from five groups from a Clayton distribution with correlation *ρ*_*i*_. The null hypothesis is *H*_0_:*ρ*_1_=0. The power is simulated with 1000 repetitions on 51 different values of *ρ*_1_ to yield a curve that is then smoothed

Figure 5 shows the power of a test of independence based on different coefficients of correlation. The dashed lines show the power of the test based only on one group of size *n*. Even though confounding would make such an operation impossible in practice, the coefficients are also calculated on the whole dataset and the power of the corresponding tests are drawn as dotted lines for reference. The mixed (dashes and dots) lines show the power of a test based on a coefficient with scalar weights. The plain lines give the power of tests based on the MAMSE-weighted sum of coefficients. The display for Kendall’s *τ* includes an additional curve with longer dashes for $\widetilde {\tau }_{{\boldsymbol {\mu }}}$.

Under the homogeneous copulas scenario, the tests based on scalar weights offer almost the same power as those using the whole dataset directly, thus illustrating Remark 2 on finite samples. With heterogeneous copulas, $\hat {\rho }_{\boldsymbol {\lambda }}$ and $\hat {\tau }_{\boldsymbol {\lambda }}$ are biased and inapplicable, but the MAMSE-based strategies offer good performances. In fact, the MAMSE-weighted coefficients seem to offer the best compromise. Under homogeneity of the copulas, they provide a test that is slightly less efficient than $\hat {\rho }_{\boldsymbol {\lambda }}$ or $\hat {\tau }_{\boldsymbol {\lambda }}$, but more efficient than the alternatives $\hat {\rho }_{1}$ or $\hat {\tau }_{1}$. If the copulas are heterogeneous across groups, then the MAMSE-weighted coefficients offer a power on par with the next best alternative: the coefficient based only on the group of interest. By adapting to the data, the MAMSE weights offer a robust alternative that gets close to the best available option without needing to know the nature of the discrepancies between groups or lack thereof.

## Conclusion

Rank statistics are used to infer the dependence structure (copula or correlation) of a distribution without estimating its marginal distributions. The presence of a discrete confounding variable may yield spurious correlations if the marginal distributions vary across the groups implied by the confounder. If the dependence structure is homogeneous across those groups, a weighted sum of the empirical copulas (or coefficients of correlation) computed from each groups provides an unbiased and asymptotically efficient solution. For heterogeneous dependence structures, we propose an adapted version of the MAMSE weights that preserves consistency while letting the groups borrow strength from each others based on the similarities of their empirical copulas. Simulations and a case study have shown that the proposed weighting schemes for rank statistics allow to account for the confounding and that although they are not uniformly more performant, the MAMSE weights provide sizable improvement in the MSE for many cases.

## Appendix

Mathematical proofs of theorems are presented below in order of appearance along with a lemma whose result is used multiple times. Proofs of trivial results are not provided.

### **Lemma 1**

Let **u**,**v**∈[0,1]^*p*^ be such that *v*_*ℓ*_≤*u*_*ℓ*_ for *ℓ*=1,…,*p*. Then

$$0\le \hat{C}_{ik}({\mathbf{u}})-\hat{C}_{ik}({\mathbf{v}}) \le \sum_{\ell=1}^{p} \frac{\lceil n_{ik}(u_{\ell}-v_{\ell})\rceil}{n_{ik}} $$

where ⌈*x*⌉ denotes the smallest integer greater or equal to *x*.

### *Proof of Lemma 1*

The lower bound is a consequence of the monotone properties of distribution functions and the relative position of **u** and **v**. For a fixed *i*, let $A^{k}_{\ell }$ be the set of points for which $R^{k}_{ij\ell }\in (v_{\ell },u_{\ell }]$. The upper bound can be derived from the probability represented by $\hat {C}_{ik}({\mathbf {u}})-\hat {C}_{ik}({\mathbf {v}})$, namely $P\left (\cup _{\ell =1}^{m} A^{k}_{\ell }\right)\le \sum _{\ell =1}^{m}P\left (A^{k}_{\ell }\right)$ following a well-known inequality. The margins being uniform on the points of the form {*a*/*n*_*ik*_:*a*=1,…,*n*_*ik*_}, we also get $P\left (A^{k}_{\ell }\right)\le \lceil n_{ik}(u_{\ell }-v_{\ell })\rceil /n_{ik}$. □

### *Proof of Theorem 3*

Consider the decomposition

$$\sup_{{\mathbf{u}}\in[0,1]^{p}}\left|\hat{C}_{{\boldsymbol{\mu}}_{k}}({\mathbf{u}}) - C_{1}({\mathbf{u}})\right|\le \sup_{{\mathbf{u}}\in[0,1]^{p}}\left|\hat{C}_{{\boldsymbol{\mu}}_{k}}({\mathbf{u}})-\hat{C}_{1k}({\mathbf{u}}) \right|+\sup_{{\mathbf{u}}\in[0,1]^{p}}\left|\hat{C}_{1k}({\mathbf{u}})- C_{1}({\mathbf{u}}) \right|. $$

The second term of the decomposition goes to 0 almost surely by the results of Deheuvels (1979), so we only need to prove that the first term does likewise. Let *ε*>0. For any given *k*∈*IN*, let **u**_*k*_=[*u*_*k*1_,…,*u*_*kp*_]^T^ be the point in [0,1]^*p*^ where $| \hat {C}_{{\boldsymbol {\mu }}_{k}}({\mathbf {u}})-\hat {C}_{1k}({\mathbf {u}})|$ is maximized. Consider the events

$$\begin{array}{@{}rcl@{}} A_{k}=\left\{\hat{C}_{1k}({\mathbf{u}}_{k})-\hat{C}_{{\boldsymbol{\mu}}_{k}}({\mathbf{u}}_{k})>\epsilon\right\}, B_{k}=\left\{\hat{C}_{{\boldsymbol{\mu}}_{k}}({\mathbf{u}}_{k})-\hat{C}_{1k}({\mathbf{u}}_{k})>\epsilon\right\}, C_{k}=\left\{{\mathbf{u}}_{k}\in\left[\frac{\epsilon}{2},1\right]^{p}\right\}. \end{array} $$

We present a proof by contradiction. If $\sup _{{\mathbf {u}}\in [0,1]^{p}}|\hat {C}_{{\boldsymbol {\mu }}_{k}}({\mathbf {u}})-\hat {C}_{1k}({\mathbf {u}}) |$ does not converge to 0, then {*A*_*k*_∪*B*_*k*_} *i*.*o*. which will happen if and only if $\left \{\left (A_{k}\cup B_{k}\cap C_{k}^{C}\right)\cup \left (A_{k}\cup B_{k}\cap C_{k}\right)\right \}\;i.o.$. We will show that neither of the two events in this decomposition can occur infinitely often.

${Case~1\!:}~A_{k}\cup B_{k}\cap C_{k}^{C}$.

We have

$$\begin{array}{@{}rcl@{}} \left| \hat{C}_{1k}({\mathbf{u}}_{k})-\hat{C}_{{\boldsymbol{\mu}}_{k}}({\mathbf{u}}_{k}) \right|\le \hat{C}_{1k}({\mathbf{u}}_{k})+\sum_{i=1}^{m} \mu_{ik} \hat{C}_{ik}({\mathbf{u}}_{k}) \le 2 \min_{\ell\in\{1,\ldots,p\}} u_{k\ell}\le \epsilon \end{array} $$

because $\hat {C}_{ik}({\mathbf {u}}_{k})>\min _{\ell \in \{1,\ldots,p\}} u_{k\ell }$ is incompatible with uniform univariate margins and the MAMSE weights sum to 1. Consequently, $A_{k}\cup B_{k}\cap C_{k}^{C}=\emptyset $ for all *k*.

*C* *a* *s* *e* 2 : *A*_*k*_∪*B*_*k*_∩*C*_*k*_.

Consider any vector **w**=[*w*_1_,…,*w*_*p*_]^T^∈[0,*ε*/(3*p*)]^*p*^. Then **u**_*k*_−**w**∈[0,1]^*p*^ since **u**_*k*_∈[*ε*/2,1]^*p*^. Next, we show that

$$\left|\hat{C}_{{\boldsymbol{\mu}}_{k}}\left({\mathbf{u}}_{k}-{\mathbf{w}}\right)-\hat{C}_{1k}\left({\mathbf{u}}_{k}-{\mathbf{w}}\right)\right|\ge \frac{\epsilon}{2}-\sum_{\ell=1}^{p} w_{\ell}\ge 0 $$

by treating two subcases. Note that the last inequality holds since *w*_*ℓ*_≤*ε*/(3*p*).

*S* *u* *b* *c* *a* *s* *e* *A* : *A*_*k*_∩*C*_*k*_.

The monotonicity of $\phantom {\dot {i}\!}C_{{\boldsymbol {\mu }}_{k}}$ and Lemma 1 allow to write

$$\begin{array}{@{}rcl@{}} \hat{C}_{1k}\left({\mathbf{u}}_{k}-{\mathbf{w}}\right)-\hat{C}_{{\boldsymbol{\mu}}_{k}}\left({\mathbf{u}}_{k}-{\mathbf{w}}\right)\ge\hat{C}_{1k}({\mathbf{u}}_{k})-\hat{C}_{{\boldsymbol{\mu}}_{k}}({\mathbf{u}}_{k})-\sum_{\ell=1}^{p} \frac{\lceil n_{1k}w_{\ell}\rceil}{n_{1k}} \ge \frac{\epsilon}{2}-\sum_{\ell=1}^{p} w_{\ell} \ge 0. \end{array} $$

as long as *k* is large enough to ensure that *p*/*n*_1*k*_<*ε*/2, and then

$$\sum_{\ell=1}^{p} \frac{\lceil n_{1k}w_{\ell}\rceil}{n_{1k}}\le \sum_{\ell=1}^{p} w_{\ell} +\frac{p}{n_{1k}}\le \frac{\epsilon}{2}+\sum_{\ell=1}^{p} w_{\ell}. $$

*S* *u* *b* *c* *a* *s* *e* *B* : *B*_*k*_∩*C*_*k*_.

By Lemma 1, we have

$$\begin{array}{@{}rcl@{}} \hat{C}_{{\boldsymbol{\mu}}_{k}}\left({\mathbf{u}}_{k}\right)-\hat{C}_{{\boldsymbol{\mu}}_{k}}\left({\mathbf{u}}_{k}-{\mathbf{w}}\right)&=&\sum_{i=1}^{m}\mu_{ik} \left\{\hat{C}_{ik}\left({\mathbf{u}}_{k}\right)-\hat{C}_{ik}\left({\mathbf{u}}_{k}-{\mathbf{w}}\right)\right\}\\ &\le& \sum_{i=1}^{m}\sum_{\ell=1}^{p} \frac{\left\lceil n_{ik}w_{\ell}\right\rceil}{n_{ik}} \le\sum_{\ell=1}^{p} w_{\ell}+\sum_{i=1}^{m}\frac{p}{n_{ik}}. \end{array} $$

For large enough values of *k*, $\sum _{i=1}^{m} p/n_{ik}<\epsilon /2$. From the previous inequality and the monotonicity of $\hat {C}_{1k}({\mathbf {u}})$, we obtain

$$\begin{array}{@{}rcl@{}} \hat{C}_{{\boldsymbol{\mu}}_{k}}\left({\mathbf{u}}_{k}\,-\,{\mathbf{w}}\right)-\hat{C}_{1k}\left({\mathbf{u}}_{k}-{\mathbf{w}}\right)\ge\hat{C}_{{\boldsymbol{\mu}}_{k}}({\mathbf{u}}_{k})-\hat{C}_{1k}({\mathbf{u}}_{k})-\sum_{\ell=1}^{p}w_{\ell}-\sum_{i=1}^{m}\frac{p}{n_{ik}}\ge \frac{\epsilon}{2}-\sum_{\ell=1}^{p}w_{\ell}\ge 0. \end{array} $$

Combining subcases A and B yields

$$\begin{array}{@{}rcl@{}} P_{k}({\boldsymbol{\mu}}_{k})&\ge&\int_{[0,1]^{p}} \left\{\hat{C}_{{\boldsymbol{\mu}}_{k}}({\mathbf{u}})-\hat{C}_{1k}({\mathbf{u}})\right\}^{2}\mathrm{d} {\mathbf{u}} \ge\int_{\left[{\mathbf{u}}_{k}-\epsilon/(3p),{\mathbf{u}}_{k}\right]^{p}} \left\{\hat{C}_{{\boldsymbol{\mu}}_{k}}({\mathbf{u}})-\hat{C}_{1k}({\mathbf{u}})\right\}^{2}\mathrm{d} {\mathbf{u}}\\ &\ge&\int_{0}^{\frac{\epsilon}{3p}}\cdots\int_{0}^{\frac{\epsilon}{3p}} \left(\frac{\epsilon}{2}-\sum_{\ell=1}^{p} w_{\ell}\right)^{2}\mathrm{d} w_{1}\cdots\mathrm{d} w_{p}=K_{p}. \end{array} $$

The number *K*_*p*_ is a fixed positive constant for any fixed *p*. As a consequence, there exists a *k*_0_ such that for all *k*≥*k*_0_, *P*_*k*_(***μ***_*k*_)>*K*_*p*_/2>0, a contradiction with Eq. 3. We must thus conclude that *A*_*k*_∪*B*_*k*_∩*C*_*k*_ occurs at most a finite number of times.

Hence, *A*_*k*_∪*B*_*k*_ occurs at most a finite number of times and $\sup _{[0,1]^{p}}\! \left |\hat {C}_{{\boldsymbol {\mu }}_{k}}\!({\mathbf {u}})\,-\, \hat {C}_{1k}({\mathbf {u}}) \right | \to \! 0$ almost surely as *k*→*∞*. □

### *Proof of Theorem 5*

It is sufficient to show that $|\int \hat {C}_{{\boldsymbol {\mu }}_{k}}({\mathbf {u}}) \mathrm {d} \hat {C}_{{\boldsymbol {\mu }}_{k}}({\mathbf {u}})-\int C({\mathbf {u}})\mathrm {d} C({\mathbf {u}})|\to 0$ almost surely as *k*→*∞*. This expression is bounded by $|\int \hat {C}_{{\boldsymbol {\mu }}_{k}}({\mathbf {u}}) \mathrm {d} \hat {C}_{{\boldsymbol {\mu }}_{k}}({\mathbf {u}})-\int C({\mathbf {u}})\mathrm {d} \hat {C}_{{\boldsymbol {\mu }}_{k}}({\mathbf {u}})|+|\int C({\mathbf {u}})\mathrm {d} \hat {C}_{{\boldsymbol {\mu }}_{k}}({\mathbf {u}})-\int C({\mathbf {u}})\mathrm {d} C ({\mathbf {u}})| $.

The first term is bounded by $\sup _{{\mathbf {u}}\in [0,1]^{p}}|\hat {C}_{{\boldsymbol {\mu }}_{k}}({\mathbf {u}})-C({\mathbf {u}}) |$ which converges to 0 almost surely by Theorem 3. For the second term, the uniform convergence in Theorem 1 implies that a sequence of random vectors with distributions $\hat {C}_{{\boldsymbol {\mu }}_{k}}({\mathbf {u}})$ will converge weakly to a random vector with distribution *C*(**u**). As a consequence, expectations of continuous bounded functions of these variables converge almost surely. □
